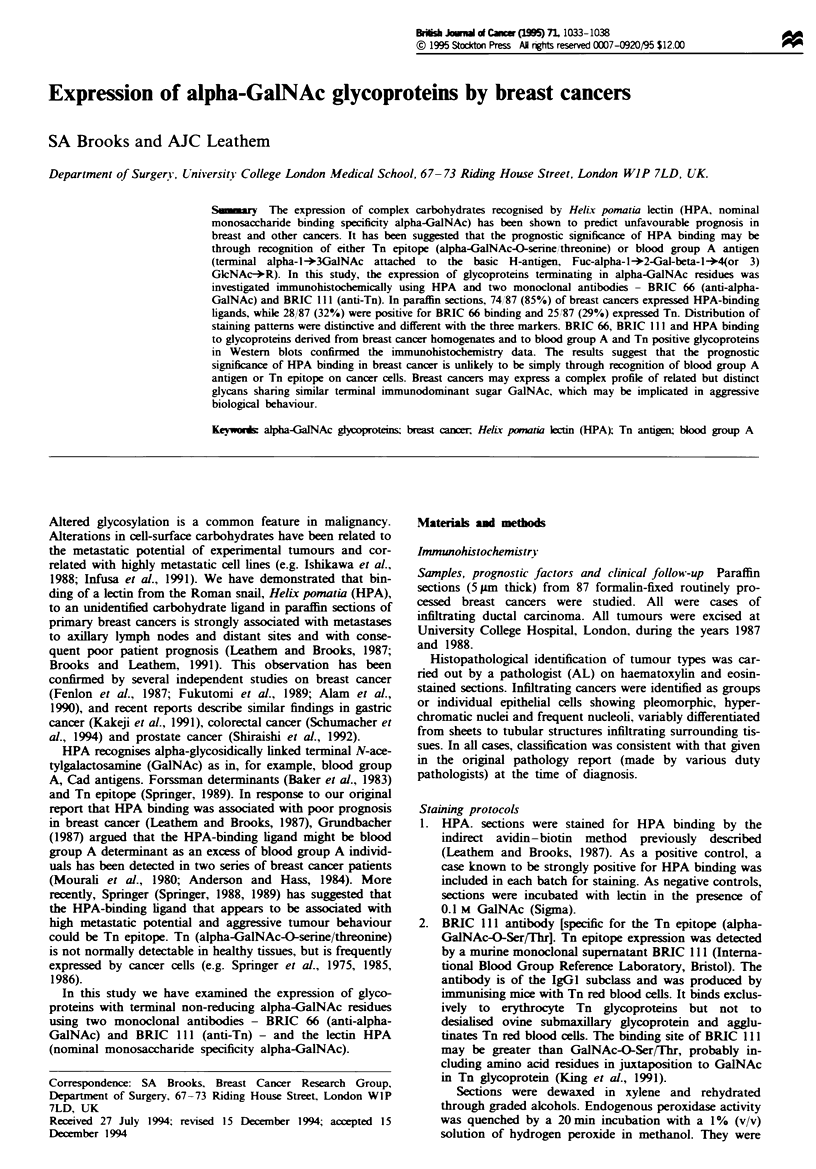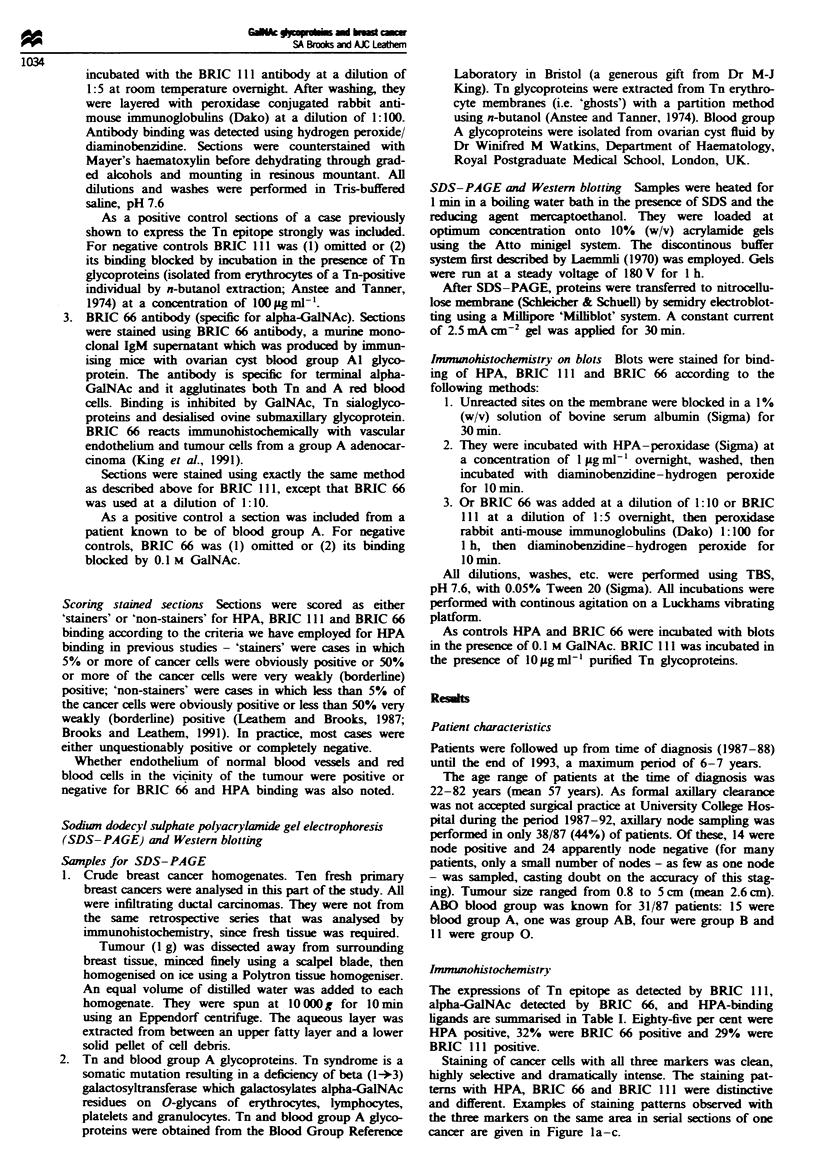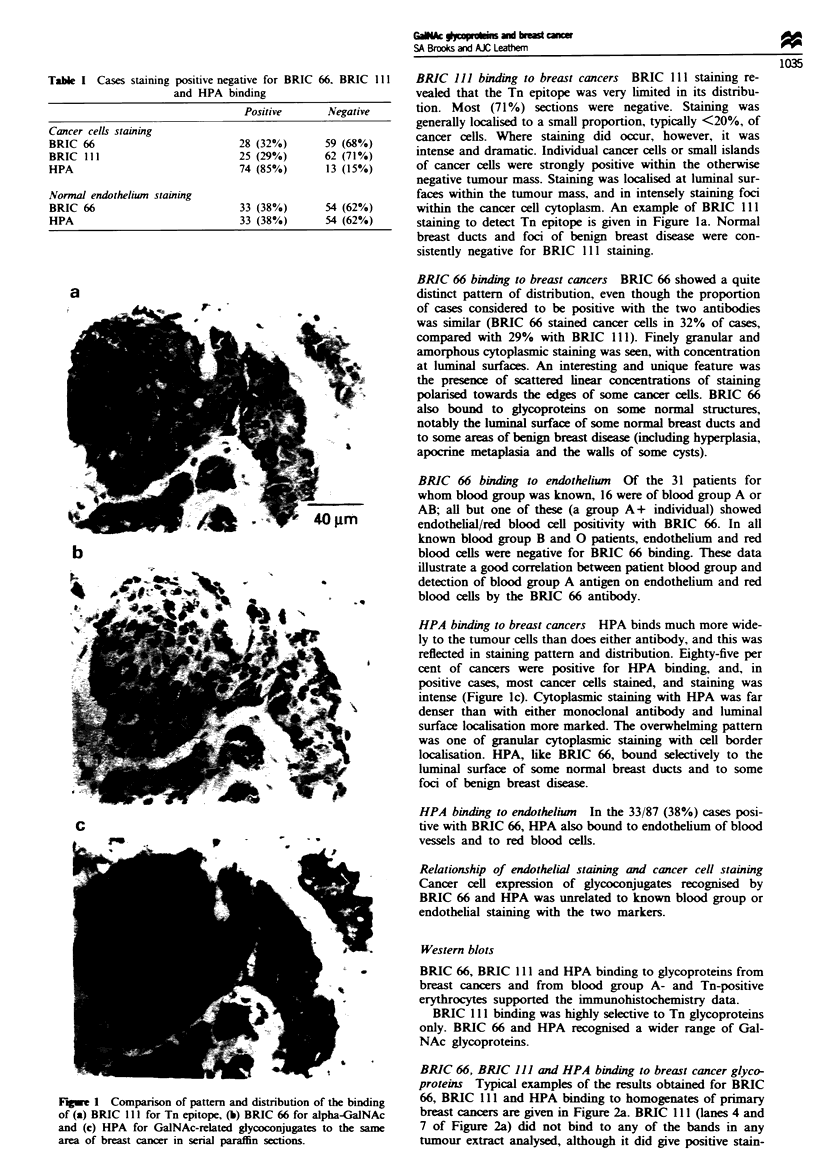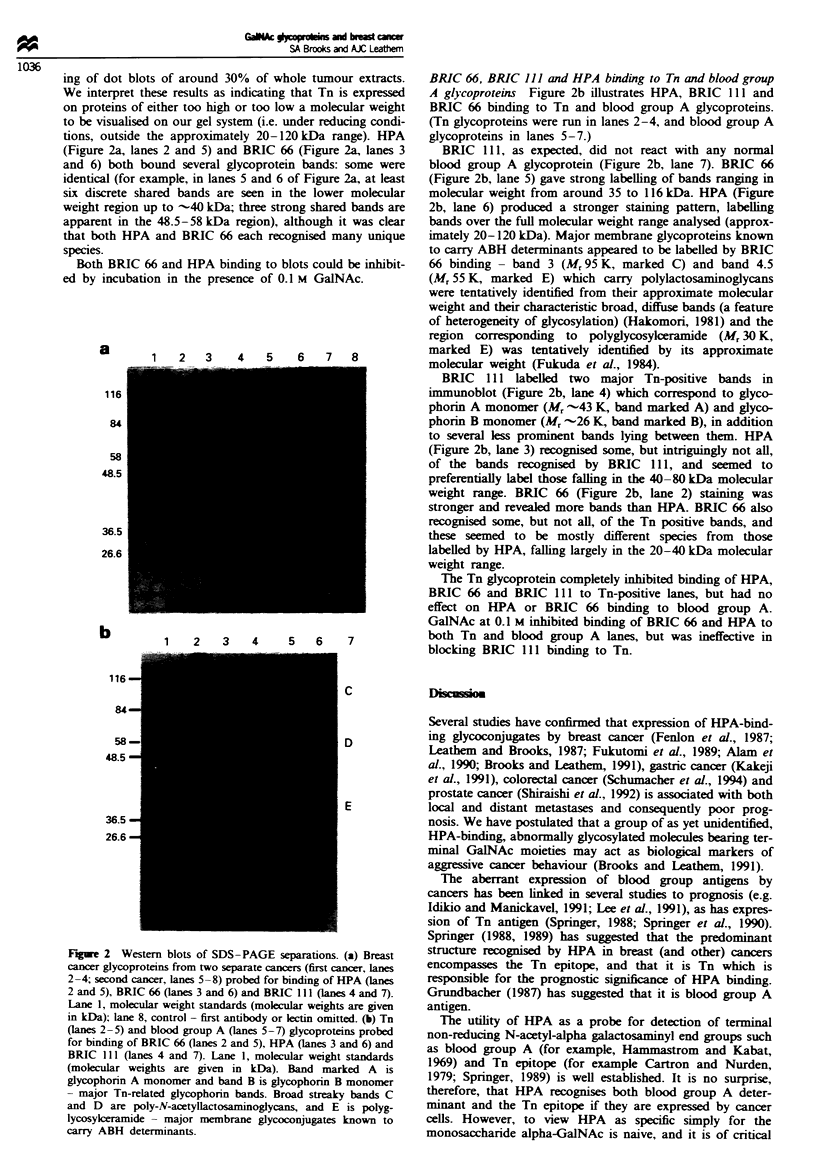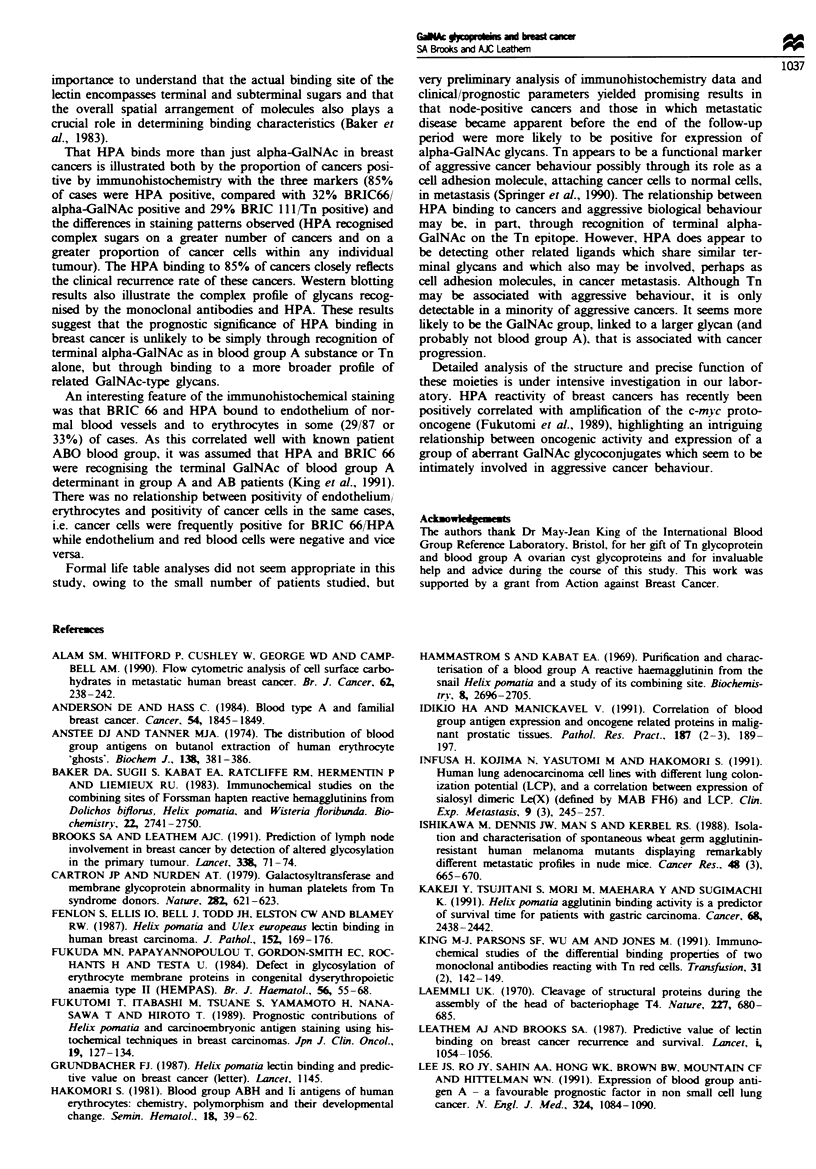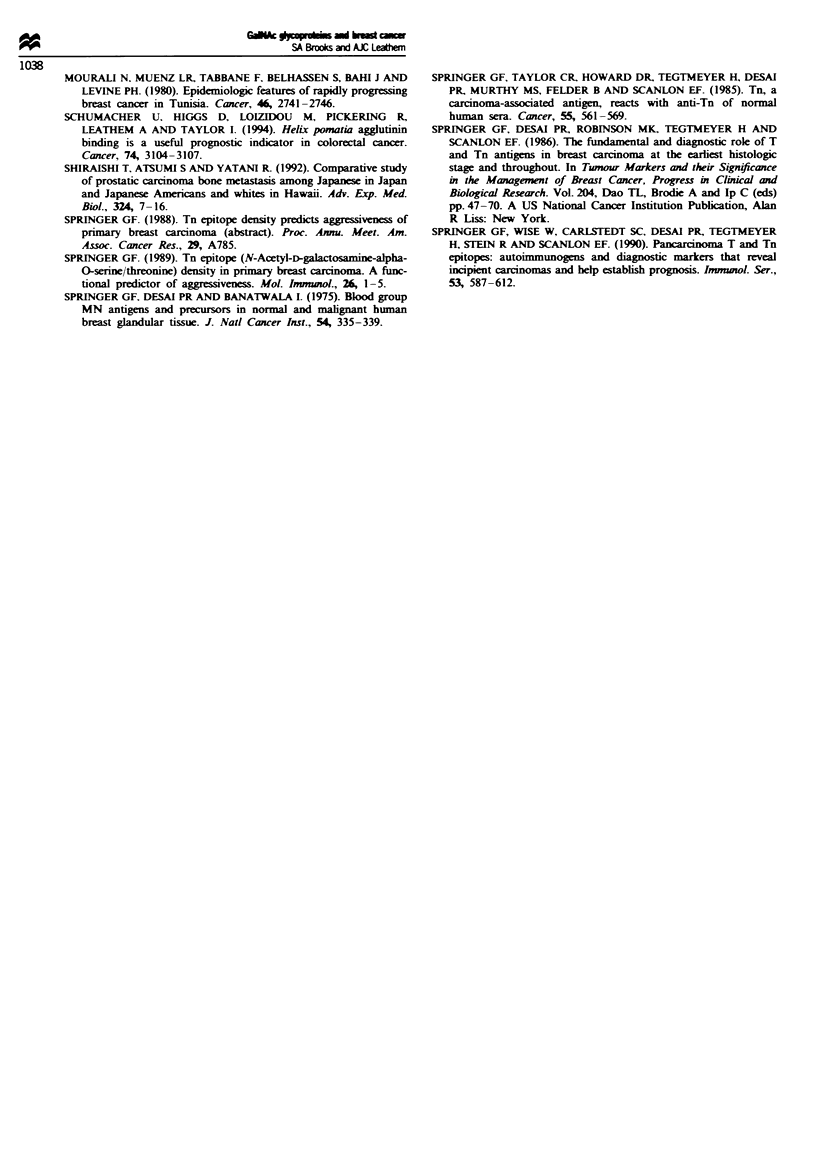# Expression of alpha-GalNAc glycoproteins by breast cancers.

**DOI:** 10.1038/bjc.1995.199

**Published:** 1995-05

**Authors:** S. A. Brooks, A. J. Leathem

**Affiliations:** Department of Surgery, University College London Medical School, UK.

## Abstract

**Images:**


British Journal of Cancer (1995) 71, 1033-1038

© 1995 Stockton Press All rights reserved 0007-0920/95 $12.00

### Expression of alpha-GalNAc glycoproteins by breast cancers

SA Brooks and AJC Leathem

*Department of Surgery, University College London Medical School, 67-73 Riding House Street, London W1P 7LD, UK.*

**Summary** The expression of complex carbohydrates recognised by *Helix pomatia* lectin (HPA, nominal monosaccharide binding specificity alpha-GalNAc) has been shown to predict unfavourable prognosis in breast and other cancers. It has been suggested that the prognostic significance of HPA binding may be through recognition of either Tn epitope (alpha-GalNAc-O-serine/threonine) or blood group A antigen (terminal alpha-1→3GalNAc attached to the basic H-antigen, Fuc-alpha-1→2-Gal-beta-1→4(or 3) GlcNAc→R). In this study, the expression of glycoproteins terminating in alpha-GalNAc residues was investigated immunohistochemically using HPA and two monoclonal antibodies - BRIC 66 (anti-alpha-GalNAc) and BRIC 111 (anti-Tn). In paraffin sections, 74/87 (85%) of breast cancers expressed HPA-binding ligands, while 28/87 (32%) were positive for BRIC 66 binding and 25/87 (29%) expressed Tn. Distribution of staining patterns were distinctive and different with the three markers. BRIC 66, BRIC 111 and HPA binding to glycoproteins derived from breast cancer homogenates and to blood group A and Tn positive glycoproteins in Western blots confirmed the immunohistochemistry data. The results suggest that the prognostic significance of HPA binding in breast cancer is unlikely to be simply through recognition of blood group A antigen or Tn epitope on cancer cells. Breast cancers may express a complex profile of related but distinct glycans sharing similar terminal immunodominant sugar GalNAc, which may be implicated in aggressive biological behaviour.

**Keywords:** alpha-GalNAc glycoproteins; breast cancer; *Helix pomatia* lectin (HPA); Tn antigen; blood group A

Altered glycosylation is a common feature in malignancy. Alterations in cell-surface carbohydrates have been related to the metastatic potential of experimental tumours and correlated with highly metastatic cell lines (e.g. Ishikawa *et al.*, 1988; Infusa *et al.*, 1991). We have demonstrated that binding of a lectin from the Roman snail, *Helix pomatia* (HPA), to an unidentified carbohydrate ligand in paraffin sections of primary breast cancers is strongly associated with metastases to axillary lymph nodes and distant sites and with consequent poor patient prognosis (Leathem and Brooks, 1987; Brooks and Leathem, 1991). This observation has been confirmed by several independent studies on breast cancer (Fenlon *et al.*, 1987; Fukutomi *et al.*, 1989; Alam *et al.*, 1990), and recent reports describe similar findings in gastric cancer (Kakeji *et al.*, 1991), colorectal cancer (Schumacher *et al.*, 1994) and prostate cancer (Shiraishi *et al.*, 1992).

HPA recognises alpha-glycosidically linked terminal *N*-acetylgalactosamine (GalNAc) as in, for example, blood group A, Cad antigens. Forssman determinants (Baker *et al.*, 1983) and Tn epitope (Springer, 1989). In response to our original report that HPA binding was associated with poor prognosis in breast cancer (Leathem and Brooks, 1987), Grundbacher (1987) argued that the HPA-binding ligand might be blood group A determinant as an excess of blood group A individuals has been detected in two series of breast cancer patients (Mourali *et al.*, 1980; Anderson and Hass, 1984). More recently, Springer (Springer, 1988, 1989) has suggested that the HPA-binding ligand that appears to be associated with high metastatic potential and aggressive tumour behaviour could be Tn epitope. Tn (alpha-GalNAc-O-serine/threonine) is not normally detectable in healthy tissues, but is frequently expressed by cancer cells (e.g. Springer *et al.*, 1975, 1985, 1986).

In this study we have examined the expression of glycoproteins with terminal non-reducing alpha-GalNAc residues using two monoclonal antibodies - BRIC 66 (anti-alpha-GalNAc) and BRIC 111 (anti-Tn) - and the lectin HPA (nominal monosaccharide specificity alpha-GalNAc).

Correspondence: SA Brooks, Breast Cancer Research Group, Department of Surgery, 67-73 Riding House Street, London W1P 7LD, UK
Received 27 July 1994; revised 15 December 1994; accepted 15 December 1994

### Materials and methods

### *Immunohistochemistry*

*Samples, prognostic factors and clinical follow-up* Paraffin sections (5 µm thick) from 87 formalin-fixed routinely processed breast cancers were studied. All were cases of infiltrating ductal carcinoma. All tumours were excised at University College Hospital, London, during the years 1987 and 1988.

Histopathological identification of tumour types was carried out by a pathologist (AL) on haematoxylin and eosin-stained sections. Infiltrating cancers were identified as groups or individual epithelial cells showing pleomorphic, hyperchromatic nuclei and frequent nucleoli, variably differentiated from sheets to tubular structures infiltrating surrounding tissues. In all cases, classification was consistent with that given in the original pathology report (made by various duty pathologists) at the time of diagnosis.

*Staining protocols*

1. HPA. sections were stained for HPA binding by the indirect avidin-biotin method previously described (Leathem and Brooks, 1987). As a positive control, a case known to be strongly positive for HPA binding was included in each batch for staining. As negative controls, sections were incubated with lectin in the presence of 0.1 M GalNAc (Sigma).
2. BRIC 111 antibody [specific for the Tn epitope (alpha-GalNAc-O-Ser/Thr]. Tn epitope expression was detected by a murine monoclonal supernatant BRIC 111 (International Blood Group Reference Laboratory, Bristol). The antibody is of the IgG1 subclass and was produced by immunising mice with Tn red blood cells. It binds exclusively to erythrocyte Tn glycoproteins but not to desialised ovine submaxillary glycoprotein and agglutinates Tn red blood cells. The binding site of BRIC 111 may be greater than GalNAc-O-Ser/Thr, probably including amino acid residues in juxtaposition to GalNAc in Tn glycoprotein (King *et al.*, 1991).

Sections were dewaxed in xylene and rehydrated through graded alcohols. Endogenous peroxidase activity was quenched by a 20 min incubation with a 1% (v/v) solution of hydrogen peroxide in methanol. They were incubated with the BRIC 111 antibody at a dilution of 1:5 at room temperature overnight. After washing, they were layered with peroxidase conjugated rabbit anti-mouse immunoglobulins (Dako) at a dilution of 1:100. Antibody binding was detected using hydrogen peroxide/diaminobenzidine. Sections were counterstained with Mayer's haematoxylin before dehydrating through graded alcohols and mounting in resinous mountant. All dilutions and washes were performed in Tris-buffered saline, pH 7.6

As a positive control sections of a case previously shown to express the Tn epitope strongly was included. For negative controls BRIC 111 was (1) omitted or (2) its binding blocked by incubation in the presence of Tn glycoproteins (isolated from erythrocytes of a Tn-positive individual by *n*-butanol extraction; Anstee and Tanner, 1974) at a concentration of $100\,\mu g\,ml^{-1}$.

3. BRIC 66 antibody (specific for alpha-GalNAc). Sections were stained using BRIC 66 antibody, a murine monoclonal IgM supernatant which was produced by immunising mice with ovarian cyst blood group A1 glycoprotein. The antibody is specific for terminal alpha-GalNAc and it agglutinates both Tn and A red blood cells. Binding is inhibited by GalNAc, Tn sialoglycoproteins and desialised ovine submaxillary glycoprotein. BRIC 66 reacts immunohistochemically with vascular endothelium and tumour cells from a group A adenocarcinoma (King *et al.*, 1991).

   Sections were stained using exactly the same method as described above for BRIC 111, except that BRIC 66 was used at a dilution of 1:10.

   As a positive control a section was included from a patient known to be of blood group A. For negative controls, BRIC 66 was (1) omitted or (2) its binding blocked by 0.1 M GalNAc.

*Scoring stained sections* Sections were scored as either 'stainers' or 'non-stainers' for HPA, BRIC 111 and BRIC 66 binding according to the criteria we have employed for HPA binding in previous studies – 'stainers' were cases in which 5% or more of cancer cells were obviously positive or 50% or more of the cancer cells were very weakly (borderline) positive; 'non-stainers' were cases in which less than 5% of the cancer cells were obviously positive or less than 50% very weakly (borderline) positive (Leathem and Brooks, 1987; Brooks and Leathem, 1991). In practice, most cases were either unquestionably positive or completely negative.

Whether endothelium of normal blood vessels and red blood cells in the vicinity of the tumour were positive or negative for BRIC 66 and HPA binding was also noted.

*Sodium dodecyl sulphate polyacrylamide gel electrophoresis (SDS–PAGE) and Western blotting*

*Samples for SDS–PAGE*

1. Crude breast cancer homogenates. Ten fresh primary breast cancers were analysed in this part of the study. All were infiltrating ductal carcinomas. They were not from the same retrospective series that was analysed by immunohistochemistry, since fresh tissue was required.

   Tumour (1 g) was dissected away from surrounding breast tissue, minced finely using a scalpel blade, then homogenised on ice using a Polytron tissue homogeniser. An equal volume of distilled water was added to each homogenate. They were spun at 10 000 *g* for 10 min using an Eppendorf centrifuge. The aqueous layer was extracted from between an upper fatty layer and a lower solid pellet of cell debris.
2. Tn and blood group A glycoproteins. Tn syndrome is a somatic mutation resulting in a deficiency of beta (1→3) galactosyltransferase which galactosylates alpha-GalNAc residues on *O*-glycans of erythrocytes, lymphocytes, platelets and granulocytes. Tn and blood group A glycoproteins were obtained from the Blood Group Reference Laboratory in Bristol (a generous gift from Dr M-J King). Tn glycoproteins were extracted from Tn erythrocyte membranes (i.e. 'ghosts') with a partition method using *n*-butanol (Anstee and Tanner, 1974). Blood group A glycoproteins were isolated from ovarian cyst fluid by Dr Winifred M Watkins, Department of Haematology, Royal Postgraduate Medical School, London, UK.

*SDS–PAGE and Western blotting* Samples were heated for 1 min in a boiling water bath in the presence of SDS and the reducing agent mercaptoethanol. They were loaded at optimum concentration onto 10% (w/v) acrylamide gels using the Atto minigel system. The discontinous buffer system first described by Laemmli (1970) was employed. Gels were run at a steady voltage of 180 V for 1 h.

After SDS–PAGE, proteins were transferred to nitrocellulose membrane (Schleicher & Schuell) by semidry electroblotting using a Millipore 'Milliblot' system. A constant current of $2.5\,mA\,cm^{-2}$ gel was applied for 30 min.

*Immunohistochemistry on blots* Blots were stained for binding of HPA, BRIC 111 and BRIC 66 according to the following methods:

1. Unreacted sites on the membrane were blocked in a 1% (w/v) solution of bovine serum albumin (Sigma) for 30 min.
2. They were incubated with HPA–peroxidase (Sigma) at a concentration of $1\,\mu g\,ml^{-1}$ overnight, washed, then incubated with diaminobenzidine–hydrogen peroxide for 10 min.
3. Or BRIC 66 was added at a dilution of 1:10 or BRIC 111 at a dilution of 1:5 overnight, then peroxidase rabbit anti-mouse immunoglobulins (Dako) 1:100 for 1 h, then diaminobenzidine–hydrogen peroxide for 10 min.

All dilutions, washes, etc. were performed using TBS, pH 7.6, with 0.05% Tween 20 (Sigma). All incubations were performed with continous agitation on a Luckhams vibrating platform.

As controls HPA and BRIC 66 were incubated with blots in the presence of 0.1 M GalNAc. BRIC 111 was incubated in the presence of $10\,\mu g\,ml^{-1}$ purified Tn glycoproteins.

### Results

*Patient characteristics*

Patients were followed up from time of diagnosis (1987–88) until the end of 1993, a maximum period of 6–7 years.

The age range of patients at the time of diagnosis was 22–82 years (mean 57 years). As formal axillary clearance was not accepted surgical practice at University College Hospital during the period 1987–92, axillary node sampling was performed in only 38/87 (44%) of patients. Of these, 14 were node positive and 24 apparently node negative (for many patients, only a small number of nodes – as few as one node – was sampled, casting doubt on the accuracy of this staging). Tumour size ranged from 0.8 to 5 cm (mean 2.6 cm). ABO blood group was known for 31/87 patients: 15 were blood group A, one was group AB, four were group B and 11 were group O.

*Immunohistochemistry*

The expressions of Tn epitope as detected by BRIC 111, alpha-GalNAc detected by BRIC 66, and HPA-binding ligands are summarised in Table I. Eighty-five per cent were HPA positive, 32% were BRIC 66 positive and 29% were BRIC 111 positive.

Staining of cancer cells with all three markers was clean, highly selective and dramatically intense. The staining patterns with HPA, BRIC 66 and BRIC 111 were distinctive and different. Examples of staining patterns observed with the three markers on the same area in serial sections of one cancer are given in Figure 1a–c.

**Table I** Cases staining positive negative for BRIC 66, BRIC 111 and HPA binding

| | *Positive* | *Negative* |
|---|---|---|
| *Cancer cells staining* | | |
| BRIC 66 | 28 (32%) | 59 (68%) |
| BRIC 111 | 25 (29%) | 62 (71%) |
| HPA | 74 (85%) | 13 (15%) |
| *Normal endothelium staining* | | |
| BRIC 66 | 33 (38%) | 54 (62%) |
| HPA | 33 (38%) | 54 (62%) |

**Figure 1** Comparison of pattern and distribution of the binding of (**a**) BRIC 111 for Tn epitope, (**b**) BRIC 66 for alpha-GalNAc and (**c**) HPA for GalNAc-related glycoconjugates to the same area of breast cancer in serial paraffin sections.

*BRIC 111 binding to breast cancers* BRIC 111 staining revealed that the Tn epitope was very limited in its distribution. Most (71%) sections were negative. Staining was generally localised to a small proportion, typically <20%, of cancer cells. Where staining did occur, however, it was intense and dramatic. Individual cancer cells or small islands of cancer cells were strongly positive within the otherwise negative tumour mass. Staining was localised at luminal surfaces within the tumour mass, and in intensely staining foci within the cancer cell cytoplasm. An example of BRIC 111 staining to detect Tn epitope is given in Figure 1a. Normal breast ducts and foci of benign breast disease were consistently negative for BRIC 111 staining.

*BRIC 66 binding to breast cancers* BRIC 66 showed a quite distinct pattern of distribution, even though the proportion of cases considered to be positive with the two antibodies was similar (BRIC 66 stained cancer cells in 32% of cases, compared with 29% with BRIC 111). Finely granular and amorphous cytoplasmic staining was seen, with concentration at luminal surfaces. An interesting and unique feature was the presence of scattered linear concentrations of staining polarised towards the edges of some cancer cells. BRIC 66 also bound to glycoproteins on some normal structures, notably the luminal surface of some normal breast ducts and to some areas of benign breast disease (including hyperplasia, apocrine metaplasia and the walls of some cysts).

*BRIC 66 binding to endothelium* Of the 31 patients for whom blood group was known, 16 were of blood group A or AB; all but one of these (a group A+ individual) showed endothelial/red blood cell positivity with BRIC 66. In all known blood group B and O patients, endothelium and red blood cells were negative for BRIC 66 binding. These data illustrate a good correlation between patient blood group and detection of blood group A antigen on endothelium and red blood cells by the BRIC 66 antibody.

*HPA binding to breast cancers* HPA binds much more widely to the tumour cells than does either antibody, and this was reflected in staining pattern and distribution. Eighty-five per cent of cancers were positive for HPA binding, and, in positive cases, most cancer cells stained, and staining was intense (Figure 1c). Cytoplasmic staining with HPA was far denser than with either monoclonal antibody and luminal surface localisation more marked. The overwhelming pattern was one of granular cytoplasmic staining with cell border localisation. HPA, like BRIC 66, bound selectively to the luminal surface of some normal breast ducts and to some foci of benign breast disease.

*HPA binding to endothelium* In the 33/87 (38%) cases positive with BRIC 66, HPA also bound to endothelium of blood vessels and to red blood cells.

*Relationship of endothelial staining and cancer cell staining* Cancer cell expression of glycoconjugates recognised by BRIC 66 and HPA was unrelated to known blood group or endothelial staining with the two markers.

### *Western blots*

BRIC 66, BRIC 111 and HPA binding to glycoproteins from breast cancers and from blood group A- and Tn-positive erythrocytes supported the immunohistochemistry data.

BRIC 111 binding was highly selective to Tn glycoproteins only. BRIC 66 and HPA recognised a wider range of GalNAc glycoproteins.

*BRIC 66, BRIC 111 and HPA binding to breast cancer glycoproteins* Typical examples of the results obtained for BRIC 66, BRIC 111 and HPA binding to homogenates of primary breast cancers are given in Figure 2a. BRIC 111 (lanes 4 and 7 of Figure 2a) did not bind to any of the bands in any tumour extract analysed, although it did give positive staining of dot blots of around 30% of whole tumour extracts. We interpret these results as indicating that Tn is expressed on proteins of either too high or too low a molecular weight to be visualised on our gel system (i.e. under reducing conditions, outside the approximately 20–120 kDa range). HPA (Figure 2a, lanes 2 and 5) and BRIC 66 (Figure 2a, lanes 3 and 6) both bound several glycoprotein bands: some were identical (for example, in lanes 5 and 6 of Figure 2a, at least six discrete shared bands are seen in the lower molecular weight region up to ~40 kDa; three strong shared bands are apparent in the 48.5–58 kDa region), although it was clear that both HPA and BRIC 66 each recognised many unique species.

Both BRIC 66 and HPA binding to blots could be inhibited by incubation in the presence of 0.1 M GalNAc.

**Figure 2** Western blots of SDS–PAGE separations. (**a**) Breast cancer glycoproteins from two separate cancers (first cancer, lanes 2–4; second cancer, lanes 5–8) probed for binding of HPA (lanes 2 and 5), BRIC 66 (lanes 3 and 6) and BRIC 111 (lanes 4 and 7). Lane 1, molecular weight standards (molecular weights are given in kDa); lane 8, control – first antibody or lectin omitted. (**b**) Tn (lanes 2–5) and blood group A (lanes 5–7) glycoproteins probed for binding of BRIC 66 (lanes 2 and 5), HPA (lanes 3 and 6) and BRIC 111 (lanes 4 and 7). Lane 1, molecular weight standards (molecular weights are given in kDa). Band marked A is glycophorin A monomer and band B is glycophorin B monomer – major Tn-related glycophorin bands. Broad streaky bands C and D are poly-*N*-acetyllactosaminoglycans, and E is polyglycosylceramide – major membrane glycoconjugates known to carry ABH determinants.

*BRIC 66, BRIC 111 and HPA binding to Tn and blood group A glycoproteins* Figure 2b illustrates HPA, BRIC 111 and BRIC 66 binding to Tn and blood group A glycoproteins. (Tn glycoproteins were run in lanes 2–4, and blood group A glycoproteins in lanes 5–7.)

BRIC 111, as expected, did not react with any normal blood group A glycoprotein (Figure 2b, lane 7). BRIC 66 (Figure 2b, lane 5) gave strong labelling of bands ranging in molecular weight from around 35 to 116 kDa. HPA (Figure 2b, lane 6) produced a stronger staining pattern, labelling bands over the full molecular weight range analysed (approximately 20–120 kDa). Major membrane glycoproteins known to carry ABH determinants appeared to be labelled by BRIC 66 binding – band 3 ($M_r$ 95 K, marked C) and band 4.5 ($M_r$ 55 K, marked E) which carry polylactosaminoglycans were tentatively identified from their approximate molecular weight and their characteristic broad, diffuse bands (a feature of heterogeneity of glycosylation) (Hakomori, 1981) and the region corresponding to polyglycosylceramide ($M_r$ 30 K, marked E) was tentatively identified by its approximate molecular weight (Fukuda *et al.*, 1984).

BRIC 111 labelled two major Tn-positive bands in immunoblot (Figure 2b, lane 4) which correspond to glycophorin A monomer ($M_r$ ~43 K, band marked A) and glycophorin B monomer ($M_r$ ~26 K, band marked B), in addition to several less prominent bands lying between them. HPA (Figure 2b, lane 3) recognised some, but intriguingly not all, of the bands recognised by BRIC 111, and seemed to preferentially label those falling in the 40–80 kDa molecular weight range. BRIC 66 (Figure 2b, lane 2) staining was stronger and revealed more bands than HPA. BRIC 66 also recognised some, but not all, of the Tn positive bands, and these seemed to be mostly different species from those labelled by HPA, falling largely in the 20–40 kDa molecular weight range.

The Tn glycoprotein completely inhibited binding of HPA, BRIC 66 and BRIC 111 to Tn-positive lanes, but had no effect on HPA or BRIC 66 binding to blood group A. GalNAc at 0.1 M inhibited binding of BRIC 66 and HPA to both Tn and blood group A lanes, but was ineffective in blocking BRIC 111 binding to Tn.

### Discussion

Several studies have confirmed that expression of HPA-binding glycoconjugates by breast cancer (Fenlon *et al.*, 1987; Leathem and Brooks, 1987; Fukutomi *et al.*, 1989; Alam *et al.*, 1990; Brooks and Leathem, 1991), gastric cancer (Kakeji *et al.*, 1991), colorectal cancer (Schumacher *et al.*, 1994) and prostate cancer (Shiraishi *et al.*, 1992) is associated with both local and distant metastases and consequently poor prognosis. We have postulated that a group of as yet unidentified, HPA-binding, abnormally glycosylated molecules bearing terminal GalNAc moieties may act as biological markers of aggressive cancer behaviour (Brooks and Leathem, 1991).

The aberrant expression of blood group antigens by cancers has been linked in several studies to prognosis (e.g. Idikio and Manickavel, 1991; Lee *et al.*, 1991), as has expression of Tn antigen (Springer, 1988; Springer *et al.*, 1990). Springer (1988, 1989) has suggested that the predominant structure recognised by HPA in breast (and other) cancers encompasses the Tn epitope, and that it is Tn which is responsible for the prognostic significance of HPA binding. Grundbacher (1987) has suggested that it is blood group A antigen.

The utility of HPA as a probe for detection of terminal non-reducing N-acetyl-alpha galactosaminyl end groups such as blood group A (for example, Hammastrom and Kabat, 1969) and Tn epitope (for example Cartron and Nurden, 1979; Springer, 1989) is well established. It is no surprise, therefore, that HPA recognises both blood group A determinant and the Tn epitope if they are expressed by cancer cells. However, to view HPA as specific simply for the monosaccharide alpha-GalNAc is naive, and it is of critical importance to understand that the actual binding site of the lectin encompasses terminal and subterminal sugars and that the overall spatial arrangement of molecules also plays a crucial role in determining binding characteristics (Baker *et al.*, 1983).

That HPA binds more than just alpha-GalNAc in breast cancers is illustrated both by the proportion of cancers positive by immunohistochemistry with the three markers (85% of cases were HPA positive, compared with 32% BRIC66/alpha-GalNAc positive and 29% BRIC 111/Tn positive) and the differences in staining patterns observed (HPA recognised complex sugars on a greater number of cancers and on a greater proportion of cancer cells within any individual tumour). The HPA binding to 85% of cancers closely reflects the clinical recurrence rate of these cancers. Western blotting results also illustrate the complex profile of glycans recognised by the monoclonal antibodies and HPA. These results suggest that the prognostic significance of HPA binding in breast cancer is unlikely to be simply through recognition of terminal alpha-GalNAc as in blood group A substance or Tn alone, but through binding to a more broader profile of related GalNAc-type glycans.

An interesting feature of the immunohistochemical staining was that BRIC 66 and HPA bound to endothelium of normal blood vessels and to erythrocytes in some (29/87 or 33%) of cases. As this correlated well with known patient ABO blood group, it was assumed that HPA and BRIC 66 were recognising the terminal GalNAc of blood group A determinant in group A and AB patients (King *et al.*, 1991). There was no relationship between positivity of endothelium/erythrocytes and positivity of cancer cells in the same cases, i.e. cancer cells were frequently positive for BRIC 66/HPA while endothelium and red blood cells were negative and vice versa.

Formal life table analyses did not seem appropriate in this study, owing to the small number of patients studied, but very preliminary analysis of immunohistochemistry data and clinical/prognostic parameters yielded promising results in that node-positive cancers and those in which metastatic disease became apparent before the end of the follow-up period were more likely to be positive for expression of alpha-GalNAc glycans. Tn appears to be a functional marker of aggressive cancer behaviour possibly through its role as a cell adhesion molecule, attaching cancer cells to normal cells, in metastasis (Springer *et al.*, 1990). The relationship between HPA binding to cancers and aggressive biological behaviour may be, in part, through recognition of terminal alpha-GalNAc on the Tn epitope. However, HPA does appear to be detecting other related ligands which share similar terminal glycans and which also may be involved, perhaps as cell adhesion molecules, in cancer metastasis. Although Tn may be associated with aggressive behaviour, it is only detectable in a minority of aggressive cancers. It seems more likely to be the GalNAc group, linked to a larger glycan (and probably not blood group A), that is associated with cancer progression.

Detailed analysis of the structure and precise function of these moieties is under intensive investigation in our laboratory. HPA reactivity of breast cancers has recently been positively correlated with amplification of the *c-myc* proto-oncogene (Fukutomi *et al.*, 1989), highlighting an intriguing relationship between oncogenic activity and expression of a group of aberrant GalNAc glycoconjugates which seem to be intimately involved in aggressive cancer behaviour.

**Acknowledgements**

The authors thank Dr May-Jean King of the International Blood Group Reference Laboratory, Bristol, for her gift of Tn glycoprotein and blood group A ovarian cyst glycoproteins and for invaluable help and advice during the course of this study. This work was supported by a grant from Action against Breast Cancer.

**References**

ALAM SM, WHITFORD P, CUSHLEY W, GEORGE WD AND CAMPBELL AM. (1990). Flow cytometric analysis of cell surface carbohydrates in metastatic human breast cancer. *Br. J. Cancer*, **62**, 238-242.

ANDERSON DE AND HASS C. (1984). Blood type A and familial breast cancer. *Cancer*, **54**, 1845-1849.

ANSTEE DJ AND TANNER MJA. (1974). The distribution of blood group antigens on butanol extraction of human erythrocyte 'ghosts'. *Biochem J.*, **138**, 381-386.

BAKER DA, SUGII S, KABAT EA, RATCLIFFE RM, HERMENTIN P AND LIEMIEUX RU. (1983). Immunochemical studies on the combining sites of Forssman hapten reactive hemagglutinins from *Dolichos biflorus, Helix pomatia*, and *Wisteria floribunda*. *Biochemistry*, **22**, 2741-2750.

BROOKS SA AND LEATHEM AJC. (1991). Prediction of lymph node involvement in breast cancer by detection of altered glycosylation in the primary tumour. *Lancet*, **338**, 71-74.

CARTRON JP AND NURDEN AT. (1979). Galactosyltransferase and membrane glycoprotein abnormality in human platelets from Tn syndrome donors. *Nature*, **282**, 621-623.

FENLON S, ELLIS IO, BELL J, TODD JH, ELSTON CW AND BLAMEY RW. (1987). *Helix pomatia* and *Ulex europeaus* lectin binding in human breast carcinoma. *J. Pathol.*, **152**, 169-176.

FUKUDA MN, PAPAYANNOPOULOU T, GORDON-SMITH EC, ROCHANTS H AND TESTA U. (1984). Defect in glycosylation of erythrocyte membrane proteins in congenital dyserythropoietic anaemia type II (HEMPAS). *Br. J. Haematol.*, **56**, 55-68.

FUKUTOMI T, ITABASHI M, TSUANE S, YAMAMOTO H, NANASAWA T AND HIROTO T. (1989). Prognostic contributions of *Helix pomatia* and carcinoembryonic antigen staining using histochemical techniques in breast carcinomas. *Jpn J. Clin. Oncol.*, **19**, 127-134.

GRUNDBACHER FJ. (1987). *Helix pomatia* lectin binding and predictive value on breast cancer (letter). *Lancet*, 1145.

HAKOMORI S. (1981). Blood group ABH and Ii antigens of human erythrocytes: chemistry, polymorphism and their developmental change. *Semin. Hematol.*, **18**, 39-62.

HAMMASTROM S AND KABAT EA. (1969). Purification and characterisation of a blood group A reactive haemagglutinin from the snail *Helix pomatia* and a study of its combining site. *Biochemistry*, **8**, 2696-2705.

IDIKIO HA AND MANICKAVEL V. (1991). Correlation of blood group antigen expression and oncogene related proteins in malignant prostatic tissues. *Pathol. Res. Pract.*, **187** (2-3), 189-197.

INFUSA H, KOJIMA N, YASUTOMI M AND HAKOMORI S. (1991). Human lung adenocarcinoma cell lines with different lung colonization potential (LCP), and a correlation between expression of sialosyl dimeric Le(X) (defined by MAB FH6) and LCP. *Clin. Exp. Metastasis*, **9** (3), 245-257.

ISHIKAWA M, DENNIS JW, MAN S AND KERBEL RS. (1988). Isolation and characterisation of spontaneous wheat germ agglutinin-resistant human melanoma mutants displaying remarkably different metastatic profiles in nude mice. *Cancer Res.*, **48** (3), 665-670.

KAKEJI Y, TSUJITANI S, MORI M, MAEHARA Y AND SUGIMACHI K. (1991). *Helix pomatia* agglutinin binding activity is a predictor of survival time for patients with gastric carcinoma. *Cancer*, **68**, 2438-2442.

KING M-J, PARSONS SF, WU AM AND JONES M. (1991). Immunochemical studies of the differential binding properties of two monoclonal antibodies reacting with Tn red cells. *Transfusion*, **31** (2), 142-149.

LAEMMLI UK. (1970). Cleavage of structural proteins during the assembly of the head of bacteriophage T4. *Nature*, **227**, 680-685.

LEATHEM AJ AND BROOKS SA. (1987). Predictive value of lectin binding on breast cancer recurrence and survival. *Lancet*, **i**, 1054-1056.

LEE JS, RO JY, SAHIN AA, HONG WK, BROWN BW, MOUNTAIN CF AND HITTELMAN WN. (1991). Expression of blood group antigen A - a favourable prognostic factor in non small cell lung cancer. *N. Engl. J. Med.*, **324**, 1084-1090.

MOURALI N, MUENZ LR, TABBANE F, BELHASSEN S, BAHI J AND LEVINE PH. (1980). Epidemiologic features of rapidly progressing breast cancer in Tunisia. *Cancer*, **46**, 2741–2746.

SCHUMACHER U, HIGGS D, LOIZIDOU M, PICKERING R, LEATHEM A AND TAYLOR I. (1994). *Helix pomatia* agglutinin binding is a useful prognostic indicator in colorectal cancer. *Cancer*, **74**, 3104–3107.

SHIRAISHI T, ATSUMI S AND YATANI R. (1992). Comparative study of prostatic carcinoma bone metastasis among Japanese in Japan and Japanese Americans and whites in Hawaii. *Adv. Exp. Med. Biol.*, **324**, 7–16.

SPRINGER GF. (1988). Tn epitope density predicts aggressiveness of primary breast carcinoma (abstract). *Proc. Annu. Meet. Am. Assoc. Cancer Res.*, **29**, A785.

SPRINGER GF. (1989). Tn epitope (*N*-Acetyl-D-galactosamine-alpha-O-serine/threonine) density in primary breast carcinoma. A functional predictor of aggressiveness. *Mol. Immunol.*, **26**, 1–5.

SPRINGER GF, DESAI PR AND BANATWALA I. (1975). Blood group MN antigens and precursors in normal and malignant human breast glandular tissue. *J. Natl Cancer Inst.*, **54**, 335–339.

SPRINGER GF, TAYLOR CR, HOWARD DR, TEGTMEYER H, DESAI PR, MURTHY MS, FELDER B AND SCANLON EF. (1985). Tn, a carcinoma-associated antigen, reacts with anti-Tn of normal human sera. *Cancer*, **55**, 561–569.

SPRINGER GF, DESAI PR, ROBINSON MK, TEGTMEYER H AND SCANLON EF. (1986). The fundamental and diagnostic role of T and Tn antigens in breast carcinoma at the earliest histologic stage and throughout. In *Tumour Markers and their Significance in the Management of Breast Cancer, Progress in Clinical and Biological Research*. Vol. 204, Dao TL, Brodie A and Ip C (eds) pp. 47–70. A US National Cancer Institution Publication, Alan R Liss: New York.

SPRINGER GF, WISE W, CARLSTEDT SC, DESAI PR, TEGTMEYER H, STEIN R AND SCANLON EF. (1990). Pancarcinoma T and Tn epitopes: autoimmunogens and diagnostic markers that reveal incipient carcinomas and help establish prognosis. *Immunol. Ser.*, **53**, 587–612.